# An 18-week model of Parent–Child Interaction Therapy: clinical approaches, treatment formats, and predictors of success for predominantly minoritized families

**DOI:** 10.3389/fpsyg.2023.1233683

**Published:** 2023-10-17

**Authors:** Jason F. Jent, William A. Rothenberg, Abigail Peskin, Juliana Acosta, Allison Weinstein, Raquel Concepcion, Chelsea Dale, Jessica Bonatakis, Cindy Sobalvarro, Felipa Chavez, Noelia Hernandez, Eileen Davis, Dainelys Garcia

**Affiliations:** ^1^Department of Pediatrics, University of Miami Miller School of Medicine, Miami, FL, United States; ^2^Center for Child and Family Policy, Duke University, Durham, NC, United States; ^3^Department of Psychology, Florida International University, Miami, FL, United States; ^4^Department of Psychiatry and Behavioral Health, The Pennsylvania State University, University Park, PA, United States; ^5^School of Psychology, Florida Institute of Technology, Melbourne, FL, United States

**Keywords:** parent–child interaction therapy, minoritized families, parent management training, child disruptive behavior, time-limited, parenting skills

## Abstract

**Introduction:**

Disruptive behavior disorders are among the most prevalent pediatric mental health referrals for young children. However, families from historically minoritized social identities have experienced disparities in treatment access, retention, and outcomes. Evidence-based interventions such as Parent–Child Interaction Therapy (PCIT) have been found to be effective in reducing children’s disruptive behaviors in minoritized families. However, variable treatment length as a result of skill-based graduation criteria (e.g., observed caregiver verbalizations) may slow and/or hinder treatment progress, particularly for families where expected treatment verbalizations are less linguistically relative (e.g., no exact English to Spanish translations) and/or culturally familiar. Time-limited PCIT has been proposed as a strategy for promoting equity in treatment completion and outcomes amongst minoritized families, because treatment progression and/ or completion is not contingent upon caregiver linguistic skill demonstration.

**Methods:**

The current study evaluated the overall effectiveness of an 18-week model of PCIT and examined predictors of retention and treatment outcomes. Participants (*N* = 488 dyads) included predominantly racially, ethnically, linguistically, and socioeconomically diverse children aged two to eight years, and their caregivers.

**Results:**

Overall findings indicate that the 18-week PCIT model is an effective intervention for reducing children’s externalizing and internalizing behaviors and improving caregiver parenting skills for most treatment completers. Despite advances in treatment completion, some caregiver social identities and PCIT treatment characteristics were predictive of lower completion rates and/or less optimal treatment outcomes.

**Discussion:**

Overall, this study provides strong support for widely disseminating use of the 18-week model of PCIT for most families served. Clinical implications and considerations for continued treatment inequity are discussed.

## 1. Introduction

Disruptive behavior disorders (DBDs) are among the most prevalent pediatric mental health referrals for young children (Polanczyk et al., 2015). Untreated behavior problems in early childhood can take a large emotional and economic toll on individuals, families, and their communities (Copeland et al., 2007; Keenan et al., 2010), and can predict behavioral, emotional, social, educational, and occupational difficulties that extend well into adulthood (Kim-Cohen et al., 2005; Munkvold et al., 2011). Disparities in prevalence of disruptive child behavior have been documented, with the highest proportions found in Black and Hispanic families (Nguyen et al., 2007; Baglivio et al., 2017). These minoritized families (i.e., families who identify as being a member of at least one social identity group that has been historically marginalized; Vaccaro and Newman, 2016) are at increased risk for experiencing inequities accross social determinants of health (e.g., food and housing, safe neighborhoods and schools, poverty, employment opportunities, and experiences of discrimination and racism), which are known to place children at higher risk for disruptive behavior and associated poor long-term outcomes. Given the substantial adverse lifelong effects of untreated disruptive behavior disorders, particularly for minoritized families, it is imperative to promote equitable access and successful engagement and completion of effective interventions.

Numerous evidence-based parent management training programs (PMTs; e.g., Incredible Years, Triple P, Parenting Management Training-Oregon, Parent–Child Interaction Therapy [PCIT]) exist that have been shown to be effective in reducing disruptive child behavior for minoritized families (Reid et al., 2001; Lau, 2006; Ortiz and Del Vecchio, 2013; Kaminski and Claussen, 2017; Garcia et al., 2021; Davis et al., 2022). Unfortunately, despite their efficacy in reducing disruptive child behavior, PMT programs have consistently documented higher attrition rates in minoritized families, indicating that significant challenges remain to successfully engaging the most vulnerable (Lundahl et al., 2006; Reyno and McGrath, 2006; Lavigne et al., 2010; Ortiz and Del Vecchio, 2013; Quetsch et al., 2020).

### 1.1. Parent–child interaction therapy

One of the most well-supported PMTs for treating child disruptive behaviors is PCIT (Thomas et al., 2017). PCIT is based on social learning theory (Patterson et al., 1982; Patterson and Fisher, 2002), employs a live coaching model, and teaches caregivers positive attention and appropriate discipline techniques to improve both child behavior and caregiver-child relationships (Eyberg and Funderburk, 2011). In the first phase of PCIT, Child-Directed Interaction (CDI), caregivers learn to follow their child’s lead in play and use skills of positive and selective attention. In the second phase of treatment, Parent-Directed Interaction (PDI), caregivers learn to use effective commands, set limits, and use appropriate discipline (time-out).

#### 1.1.1. Standard criteria-based PCIT protocol

The standard PCIT protocol (Eyberg and Funderburk, 2011) is “criteria-based,” requiring caregivers to demonstrate a minimum set of parenting skills, which include using “Do” skills (i.e., labeled praise, behavior descriptions, reflections) and reducing “Avoid” skills (i.e., questions, commands, critical statements) to move from the CDI phase to the PDI phase of treatment. Caregivers must also show proficiency in the effective use of commands and consistency in follow-through, as well as rate the child’s disruptive behaviors within normal limits in order to meet “graduation criteria” or complete treatment (Eyberg and Funderburk, 2011). Within criteria-based PCIT, time to treatment completion varies widely and depends on a complex set of factors involving not only caregiver skill acquisition and child progress, but also practical barriers such as the cultural and linguistic (un)fit of the intervention and related undetermined amount of time until graduation/service completion/treatment termination (Herschell et al., 2018; Ramos et al., 2018). Assuming that caregivers attend 60% of scheduled sessions (Comer et al., 2017), criteria-based PCIT can take an average of 21–36 weeks to complete, which is a significant and likely strenuous time commitment, particularly for minoritized families. Similar to treatment length, there is wide variability in attrition rates for criteria-based PCIT, ranging from 32% to 73%, with higher rates of attrition in community mental health settings serving predominantly minoritized families (McCabe and Yeh, 2009; Lyon and Budd, 2010; Lanier et al., 2011; Abrahamse et al., 2016; Quetsch et al., 2020).

#### 1.1.2. Alternative models: time-limited and session-limited PCIT

To better engage families, alternative models of PCIT have been developed that may address limitations of criteria-based PCIT. These models, known as “time-limited” or “session-limited” PCIT, do not require demonstrating skill proficiency or specific child behavior ratings. Instead, they are structured based on a predetermined length of time or a set number of sessions. In a meta-analysis that examined different formats of PCIT, time-limited and session-limited PCIT were shown to effectively reduce children’s disruptive behaviors to within normal limits as well as improve retention rates in some instances (Thomas et al., 2017). Given the success of time- and session-limited PCIT models, further exploration of these models is warranted to determine how these models may impact retention and treatment outcomes, particularly for minoritized families.

### 1.2. PCIT models: criteria-based vs. time- and session-limited effectiveness

While all PCIT models have demonstrated large effect sizes for reducing child disruptive behavior, criteria-based PCIT has been associated with the largest reductions in disruptive behaviors relative to time- and session-limited PCIT (Thomas et al., 2017). This, in part, is to be expected given that meeting “graduation criteria” is contingent upon caregivers rating child behaviors within a half standard deviation of the mean on a measure of child disruptive behavior (i.e., Eyberg Child Behavior Inventory; Eyberg and Pincus, 1999). Nonetheless, several PCIT studies have shown that many caregivers rate their children as meeting or nearly meeting graduation criteria for disruptive behavior by the end of the first phase of treatment (CDI; e.g., Stokes et al., 2016; Comer et al., 2017; Jent et al., 2021). These findings call into question the additional value of requiring families to meet parenting skill proficiency for treatment graduation when the family’s primary presenting problem, child disruptive behavior, is no longer clinically elevated.

### 1.3. Structural aspects of PCIT and minoritized families

Questions surrounding the additional value of requiring parenting skill criteria to move from CDI to PDI and complete treatment are especially relevant when considering structural aspects of PCIT that possibly impact family engagement and outcomes for minoritized families. Family identification as being a member of a minoritized social identity group has been a persistent predictor of lower treatment attendance and skill acquisition (Lavigne et al., 2010). PCIT studies have shown that minoritized families from under-resourced communities require more treatment sessions to meet PCIT skill proficiency (Fernandez and Eyberg, 2009; Matos et al., 2009; McCabe and Yeh, 2009) than higher-income, White caregivers, These findings are possibly due to targeted verbal skills taught and reinforced in PCIT being perceived as less culturally familiar or acceptable (McCabe and Yeh, 2009; Lau et al., 2011; Ramos et al., 2018). Further, although Spanish- and English-speaking caregivers increase their use of “Do” skills and decrease their use of “Avoid” skills at similar rates, Spanish-speaking families use significantly more “Avoid” skills than their English-speaking counterparts (Matos et al., 2009; Ramos et al., 2018). While this finding has been previously theorized to reflect a desire to inculcate values of respect and obedience to authority within Hispanic families (Calzada et al., 2012; Ramos et al., 2018), it also may lead to inequities in treatment progression (i.e., time for families to transition from CDI to PDI) and treatment completion when caregivers are naturally starting at a higher level of “Avoid” skills but experiencing similar rates of skills acquisition. Slower progression in treatment is particularly concerning for minoritized families given pervasively documented disparities in access to services and inherent systemic external stressors (e.g., poverty, community violence, racism) that may exacerbate behavioral and emotional difficulties in children (Abe-Kim et al., 2007; Fowler et al., 2009; Alegria et al., 2010). These findings suggest that there may be some aspects of PCIT that do not address the needs of minoritized families or may not align with their cultural values, beliefs, and practices.

### 1.4. PCIT engagement strategies

To date, numerous strategies, including cultural adaptations, culturally informed assessments, structural barrier reductions, and alternative treatment models, have been examined with the goal of increasing PMT engagement with minoritized families yielding mixed results. Cultural adaptations of PMTs (McCabe and Yeh, 2009) and low-cost incentives (Quetsch et al., 2020) have been shown to yield high satisfaction but limited gains in treatment engagement. Fortunately, other strategies including incorporating brief cultural assessment (i.e., Cultural Formulation Interview; American Psychiatric Association, 2013) within PCIT assessment procedures (Sanchez et al., 2021), providing simultaneous ancillary support services (i.e., Natural Helpers) into PCIT delivery (Davis et al., 2022; Garcia et al., 2023), and delivering PCIT services virtually (i.e., Internet-delivered PCIT [I-PCIT]; Comer et al., 2017; Garcia et al., 2021; Peskin et al., 2023), have all shown promising findings with regard to overcoming structural barriers to care (e.g., transportation, difficulties navigating healthcare systems; Derr, 2016) and personalizing services to better engage minoritized families in PCIT.

Although recent efforts have been made to develop guidelines for culturally adapting parenting interventions, such as MYPCIT (a culturally personalized approach to PCIT; McCabe et al., 2020), the PCIT treatment graduation criteria was originally developed for English speakers, making it inherently biased toward English-language connotations. For example, the connotation of commands like “Mira” (“Look”) and “Dale” (“Go ahead”/“Do it”/“Give it”) used in Spanish do not have the same connotation as their direct translation into English. Further, the behavior observation system that is used for determining PCIT skill proficiency has been normed and validated with predominantly English-speaking and White caregivers (Thornberry and Brestan-Knight, 2011; Eyberg et al., 2013; Cotter and Brestan-Knight, 2020), placing undue burden on PCIT therapists delivering services in languages other than English to make decisions about caregiver skill proficiency when direct translations are not available or when typical phrasing of statements are dissimilar between two languages. For example, a recent study highlighted differences in PCIT coaching statements, with therapists delivering services in Spanish using more coaching statements than therapists delivering services in English, which may in part be due to the need for additional context and clarification for aspects of PCIT that do not generalize well to the Spanish language (Green Rosas et al., 2022).

Imposing additional “roadblocks” to completing PCIT through possibly culturally-biased determination of progress is unproductive to the goal of improving behavioral health outcomes in families who need it the most. Therefore, time-limited or session-limited PCIT, in which treatment progression is not solely based on families demonstrating skill criteria, may offer a more equitable approach to PMT for minoritized families (Sanders et al., 2014; Tully and Hunt, 2017). There is a need to better understand whether engagement and retention strategies, such as session-limited or time-limited models of PCIT, preserve effective treatment outcomes for minoritized families to move closer to equity in service delivery (Hoffman and Markovits, 2022).

### 1.5. The current study

The current study investigated the extent to which a time-limited (i.e., 18-week) model of PCIT was predictive of increased equity in treatment engagement and family outcomes for predominantly minoritized families in a large metropolitan area in the Southeast United States.

The time-limited model was selected based on a review of historical criteria-based PCIT clinic data highlighting higher rates of attrition for caregivers who spoke Spanish, had lower levels of education, and/or identified as Black or African American (Rothenberg et al., 2019; Jent et al., 2021). Within said reviews, length of treatment was also found to be longer for Spanish-speaking families primarily due to difficulties meeting parenting skill proficiency. The 18-week model of PCIT described in the current study was designed to be responsive to these findings by providing families a minimum of 12 sessions (projected minimum 70% attendance rate), similar to other session-limited models (Bagner and Eyberg, 2007; Bagner et al., 2010; Webb et al., 2017). Further, the current study also includes strategies shown to positively impact treatment engagement for minoritized families, such as incorporating an adapted version of the Cultural Formulation Interview (i.e., CFI; American Psychiatric Association, 2013) to the pre-treatment assessment, which provides guidance to therapists on cultural factors that might impact treatment (Sanchez et al., 2021). Additionally, services were offered in additional treatment settings (i.e., neighborhood-embedded clinics; Davis et al., 2022) and virtual services (I-PCIT; Garcia et al., 2021) and formats (i.e., the addition of Natural Helpers; Garcia et al., 2023). Nonetheless, the extent that these strategies impact treatment engagement and outcomes within a time-limited model is unknown.

Overall, the main goal of the current study was to examine who may benefit from an 18-week model of PCIT regarding treatment retention and family outcomes. First, we examined how the 18-week model impacted child outcomes (i.e., externalizing behaviors, internalizing behaviors, and compliance) and caregiver outcomes (i.e., parenting skills) and the extent to which the 18-week model of PCIT produced clinically significant changes in child externalizing and internalizing behaviors (i.e., child behavior within normal limits at post and follow-up). Then, we examined whether there are specific family and treatment factors (e.g., sociodemographic, treatment format-related, pre-treatment variables, treatment process variables) that predict improvements in treatment retention (i.e., completion of the first phase of treatment [CDI] and/or completion of treatment) and child and caregiver outcomes over the course of treatment (i.e., mid-treatment, post-treatment, 1-month follow-up). An understanding of the effectiveness of time-limited PCIT for predominantly minoritized families may be helpful for increasing clinicians’ understanding for whom a shorter duration of PCIT may be most beneficial, relative to the criteria-based PCIT protocol. In addition, findings may provide a better understanding of where time-limited PCIT falls short in promoting treatment equity.

## 2. Materials and methods

### 2.1. Participants

Participating children (*N* = 488) aged 2–8 years (*M* = 4.72, SD 1.55; 70% assigned male at birth) and their primary caregivers (92.26% female) received PCIT services at six university-affiliated clinics or virtually (I-PCIT). Two treatment sites were located in academic centers, while the remaining four clinics were embedded within local communities in a large metropolitan area in the Southeast United States. All treatment was grant-funded and therefore free to participating families. Demographic information was collected through an online database management system, REDCap (Harris et al., 2009), wherein caregivers self-selected responses. For additional demographic characteristics, see Table 1.

**Table 1:** Sample descriptive statistics.

	***M* or %**	***SD***
Child gender (% Male)	70.12%	--
Child age (Months)	56.63	18.58
Caregiver education		
Some Grade School	4.82%	
High School Diploma/GED	11.11%	
Some College	15.72%	
Associate’s Degree	10.69%	
Bachelor’s Degree	27.25%	
Graduate Degree	30.40%	
HUD Low-Income (% Low Income)	49.58%	--
Languages PCIT services provided in		
English	66.53%	
Spanish	25.05%	
English and Spanish	7.60%	
Creole and English	0.82%	
Parent gender (% Female)	92.26%	--
% Families Where 2 Caregivers Participating	30.45%	--
Randomized to Natural Helper Intervention	10.45%	--
Received Cultural Formulation Interview	52.05%	--
Participated During COVID-19	53.31%	--
Completed PCIT *via* telehealth	51.02%	--
Pretreatment PSI Total Stress Percentile	64.3	22.79
Completed Child Directed Interaction Phase	75.41%	--
Completed Entire PCIT Treatment	65.57%	--
Caregiver Ethnicity: % Hispanic	67.57%	--
	Hispanic (%)	Not Hispanic (%)
Caregiver Race: Native American	0.63%	0%
Caregiver Race: Asian	0.21%	1.05%
Caregiver Race: Black	1.26%	9.00%
Caregiver Race: Pacific Islander	0.21%	0%
Caregiver Race: White	53.12%	18.20%
Caregiver Race: Other	6.07%	0.42%
Caregiver Race: Multiracial	6.07%	1.67%
Caregiver Race: Haitian or English-Speaking Caribbean	0%	2.09%

HUD, Housing and Urban Development Criteria; PCIT, Parent-Child Interaction Therapy.

Study inclusion criteria consisted of (a) the child being between the ages of 2 and 8 years, (b) the primary caregiver being fluent in English, Spanish, or Creole, and (c) elevated child disruptive behavior on the Eyberg Child Behavior Inventory Intensity Scale (ECBI; Intensity Raw Score ≥ 131; Eyberg and Pincus, 1999) or on the Externalizing Problems subscales or composite of the Behavior Assessment System for Children, Third Edition (BASC-3; T-Score ≥ 60; Reynolds and Kamphaus, 2015) or a history of involvement with child protective services. If the family did not meet the inclusion criteria, they were provided referrals and resources to other services within the community. Institutional Review Board (IRB) approval was obtained from the university and all participants who agreed to be in the study signed an informed consent. All study procedures were conducted in accordance with the ethical standards of the IRB.

### 2.2. Study design and procedures

#### 2.2.1. Assessments

If inclusion criteria were met following screening, families were contacted by a PCIT therapist to schedule an intake session (i.e., pre-treatment assessment), which consisted of obtaining written consent (and child assent, for children ages 7 years or older) and completion of a clinical interview (standard interview or adapted CFI; American Psychiatric Association, 2013) and a 20-min behavioral observation. Electronic caregiver-report questionnaires about child and family functioning were sent out following this session.

Of the 488 families enrolled, 476 completed a pre-treatment assessment (97.5%), 372 completed a mid-treatment assessment (76.23%), 330 families completed a post-treatment assessment (67.62%), and 236 families completed a 1-month follow-up assessment (48.36%). During the post-treatment assessment, families completed treatment outcome and satisfaction questionnaires in addition to the 20-min behavioral observation administered at pre-treatment. At the 1 month follow-up, families completed treatment outcome questionnaires and the 20-min behavioral observation.

#### 2.2.2. PCIT

Families received weekly, one-hour individual sessions with a PCIT therapist across an 18-week (time-limited) period. Therapists coached caregivers on the newly learned parenting skills *in-vivo* from behind a one-way mirror via a wireless headset or via telehealth. The telehealth approach was completed using the Zoom for Healthcare platform. Tablets, Bluetooth headsets, and toys were provided for families who needed equipment. Master’s and doctoral level therapists (*N* = 24, 87.5% Female, 50% White Hispanic, 8% Black or African American, 50% English/Spanish bilingual, 4% English/Creole bilingual) with backgrounds in clinical psychology and mental health counseling delivered the time-limited PCIT protocol. The treatment protocol consisted of two pre-treatment assessments (e.g., clinical interview and behavioral observation), CDI and PDI didactic sessions, a maximum of 5 CDI coach sessions, and the remaining weeks committed to PDI coach sessions. Families could progress through CDI in fewer sessions if they met skill proficiency (i.e., 10 labeled praises, 10 reflections, 10 descriptions, and 3 or fewer questions, commands, and criticisms). Families were able to graduate in fewer than 18 weeks if they met graduation criteria [i.e., CDI (see above) and PDI skill proficiency (75% effective command rate and 75% correct follow through), ECBI Intensity raw score < = 114, and caregiver reported confidence in their ability to manage their child’s behaviors; Eyberg and Funderburk, 2011). Appointments were scheduled Monday through Thursday with Friday allotted for make-up sessions as needed. Therapists received PCIT training and weekly supervision with a Certified PCIT International Within Agency or Regional Trainer.

##### 2.2.2.1. PCIT + Natural Helper

Within three community-based satellite clinics, families were provided the opportunity to also receive services from Natural Helpers in addition to PCIT. Natural Helpers are lay community health workers trained to help increase treatment engagement and participation within predominantly low-income and marginalized communities (Barnett et al., 2016). Natural Helpers were recruited from the communities surrounding the PCIT clinics. They received seven, four-hour sessions of training upon hire (Barnett et al., 2016). As the Natural Helpers worked with families, they participated in weekly consultation with the PCIT therapists to discuss family progress and to problem-solve barriers. Natural Helpers also attended bi-weekly group implementation meetings to discuss recruitment strategies, complete skill and coding practice exercises, review session videos, and address logistical concerns.

Beyond PCIT service delivery by therapists, Natural Helpers worked with families via home-based visits or telehealth sessions to (1) explain parenting principles in a culturally responsive manner (e.g., navigating intergenerational parenting within the same household); (2) support caregivers in their skill acquisition (e.g., observing in-home caregiver-child interactions, providing strength-based feedback, and engaging in role plays to practice skills being learned); and (3) troubleshoot structural barriers to services and other basic life needs (e.g., transportation, food and housing assistance, work or immigrant visa, and asylum navigation).

### 2.3. Measures

#### 2.3.1. Child externalizing and internalizing behavior outcomes

The ECBI is a 36-item caregiver-report measure of disruptive behaviors in children ages 2–16 years (Eyberg and Pincus, 1999). The Intensity Scale assesses the frequency of behavior problems, with a raw score of 131 or higher (T-score > = 60) indicating clinically elevated frequency of disruptive behavior. The ECBI has demonstrated acceptable levels of reliability (α = 0.94, test–retest = 0.75; Gross et al., 2007), and validity (e.g., positive association with other measures of externalizing behavior and emotion dysregulation; Rothenberg et al., 2019; Davis et al., 2022; Garcia et al., 2023) in racially and ethnically diverse populations, stability over time, and sensitivity to treatment change. The ECBI Intensity Scale was used for eligibility and as an outcome measure of child externalizing behavior.

The Behavior Assessment System for Children, Third Edition, Parent Rating Scale (BASC-3 PRS; Reynolds and Kamphaus, 2015) is a caregiver-report measure of emotional, behavioral, and adaptive functioning of children ages 2–21 years, in which caregivers rate the frequency of behaviors. The BASC-3 PRS has well-established reliability (*α* = 0.83–0.96, test–retest = 0.87–0.92; Reynolds and Kamphaus, 2015) and validity in racially and ethnically diverse samples (e.g., positive association with other measures of externalizing behavior and emotion dysregulation; Rothenberg et al., 2019; Davis et al., 2022; Garcia et al., 2023). The BASC-3 PRS Externalizing Problem Composite was used for eligibility and as an outcome, the Externalizing and Internalizing Problems Composites were used as measures of child externalizing and internalizing behaviors.

#### 2.3.2. Parenting skills

An observational assessment method was used to evaluate parenting skills. The Dyadic Parent–Child Interaction Coding System, Fourth Edition (DPICS-IV; Eyberg et al., 2013) is a behavioral observation tool used to code and evaluate caregiver verbalizations, behaviors, and the quality of caregiver and child interactions. PCIT therapists trained to 80% coding interrater agreement on the DPICS-IV coded the three 5-min parent–child observations: (1) child-led play (CLP), in which the caregiver is instructed to follow the child’s lead in the play, (2) caregiver or parent-led play (PLP), in which the caregiver is instructed to select the play activity, and (3) clean-up (CU), in which the child is instructed to clean up the play activity independently. The frequency of “Do” skills and “Avoid” skills were coded during CLP observations. The DPICS-IV demonstrates acceptable reliability and validity of the coding categories in English-speaking populations (Eyberg et al., 2014). In the current study, the DPICS-IV was used as an outcome measure of caregiver skill acquisition. Specifically, a composite score of “Do” skills (sum of Labeled Praises, Reflections and Descriptions) and “Avoid” skills (sum of Questions, Commands and Negative Talk) were created. Child compliance was coded during five-minute caregiver-led play and clean-up situations in which the percentage of child compliance to caregiver effective commands were calculated.

#### 2.3.3. Family and treatment characteristics

Caregivers reported information about their family *via* an online survey including child sex assigned at birth, child age, caregiver education, caregiver race and ethnicity, household income, and household size. Household income and household size were utilized to code families into either being within or above the low-income limits for the region as defined by the United States Housing and Urban Development (HUD) for FY2022. This was coded as a binary variable (1 = within low-income limits, 0 = above low-income limits). Therapists also reported the number of caregivers who participated in PCIT and the language that services were delivered in (English, Spanish, English and Spanish, English and Creole). Parenting stress was measured using the Parenting Stress Index: Short Form (4th Edition) Total Stress percentile score (Abidin, 2012). Other treatment characteristics were coded as dichotomous variables for the current study (1 = yes, 0 = no) including whether: families received the Cultural Formulation Interview as a part of their initial assessment; services were completed during COVID-19 (1 = Services completed between March 2020–August 2022, 0 = Services completed between February 2018–February 2020), services were completed virtually; and/or families were randomized to the Natural Helper intervention.

#### 2.3.4. Treatment retention

In the current study, attending five sessions or meeting CDI skill proficiency (see *PCIT* section) at or before CDI Coach session five was defined as CDI completion. Treatment completion was defined as reaching 18 weeks of treatment or meeting PCIT graduation criteria (see *PCIT* section).

### 2.4. Data analytic plan

Study analyses proceeded in a series of sequential steps in accordance with best practices to address each study aim (Kline, 2011; McDonald, 2014). First, we analyzed how well the 18-week model worked to change the child (i.e., externalizing and internalizing behaviors, child compliance) and caregiver (i.e., parenting skills) outcomes. To do so, we conducted paired samples t-tests that examined whether changes from pre-treatment to post-treatment and from pre-treatment to follow-up on these measures were statistically significant (McDonald, 2014). Importantly, critical value of *p*s in these planned comparisons were Bonferroni-adjusted to protect against Type-I error inflation that may emerge due to making 2 comparisons (pre-treatment to post-treatment and pre-treatment to follow-up) per measure (Bonferroni, 1936). See Tables 2, 3 for further detail.

**Table 2:** Changes in key parenting and child outcome variables over time in 18-week model.

	Pre-Treatment			Post-Treatment			1-Month Follow-Up			Pre-Post Comparison			Pre-Follow-Up Comparison		
Variable	*N*	*M*	*SD*	*N*	*M*	*SD*	*N*	*M*	*SD*	*t*	*p*	*d*	*t*	*p*	d
ECBI	466	146.28	31.09	315	89.28	29.11	217	92.71	28.76	**31.7**	**<0.01**	**1.79**	**25.16**	**<0.01**	**1.71**
% Child compliance	441	52.93	40.26	284	74.61	36.65	204	74.08	34.52	**−7.64**	**<0.01**	**−0.46**	**−5.94**	**<0.01**	**−0.42**
BASC-Ext	321	63.12	12.07	201	52.26	9.44	N/A	N/A	N/A	**17.16**	**<0.01**	**1.24**	N/A	N/A	N/A
BASC-Int	320	56.62	11.77	192	49.38	9.38	N/A	N/A	N/A	**11.51**	**<0.01**	**0.85**	N/A	N/A	N/A
“Do” skills	459	4.74	4.42	289	26.80	11.84	209	24.34	10.93	**−32.20**	**<0.01**	**−1.90**	**−26.28**	**<0.01**	**−1.82**
“Avoid” skills	459	28.88	16.56	289	5.68	6.74	209	6.22	6.71	**24.02**	**<0.01**	**1.42**	**20.47**	**<0.01**	**1.42**

ECBI, Eyberg Child Behavior Inventory Intensity Scale Raw Score; BASC-Ext, Behavioral Assessment System for Children, Third Edition Externalizing Problems Composite *t*-score, BASC-Int, Behavioral Assessment System for Children, Third Edition Internalizing Problems Composite *t*-score. N/A, Data was not collected on that variable at that time point. Bolded values indicate tests that were significant at *p* < 0.01. Notably, all effects are statistically significant even after making the Bonferroni correction to adjust the value of *p* to protect against Type I error inflation. The Bonferroni adjusted critical value of *p* for each variable here would be 0.05/2 = 0.025 (because we are making 2 comparisons for each variable: Pre-treatment to Post-treatment and Pre-Treatment to 1-Month Follow-Up). All *p*-values reported here are still below this critical value of *p*.

**Table 3:** Clinical significance of child outcomes at post-treatment and the 1-month follow-up.

Criterion	Pre-treatment	Post-treatment	1-Month follow-up	Pre-treatment-post-treatment comparison		Pre-treatment-1-month-follow up comparison	
				McNemar’s χ^2^	*p*	McNemar’s χ^2^	*p*
% of Sample Within Normal Limits on the ECBI	29.61%	92.70%	89.86%	**198.00**	**<0.01**	**127.03**	**<0.01**
% of Sample Hitting Graduation Criteria on the ECBI	14.59%	83.49%	78.80%	**217.02**	**<0.01**	**135.03**	**<0.01**
% of Sample Within Normal Limits on the BASC-Ext	42.06%	81.59%	N/A	**79.00**	**<0.01**	N/A	N/A
% of Sample Within Normal Limits on the BASC-Int	65.00%	84.38%	N/A	**29.88**	**<0.01**	N/A	N/A

ECBI, Eyberg Child Behavior Inventory Intensity Scale Raw Score; BASC-Ext, Behavioral Assessment System for Children, Third Edition Externalizing Problems Composite *t*-score; BASC-Int, Behavioral Assessment System for Children, Third Edition Internalizing Problems Composite *t*-score. N/A, Data was not collected on that variable at that time point. Bolded values indicate tests that were significant at *p* < 0.01. Notably, all effects are statistically significant even after making the Bonferroni correction to adjust the critical *p*-value to protect against Type I error inflation. The Bonferroni adjusted critical value of *p* for each variable here would be 0.05/2 = 0.025 (because we are making 2 comparisons for each variable: Pre-treatment to Post-treatment and Pre-Treatment to 1-Month Follow-Up). All value of *p*s reported here are still below this critical value of *p*.

Second, we evaluated the extent to which the 18-week model of PCIT produced clinically significant changes in child externalizing and internalizing behaviors. Consistent with prior PCIT studies (Comer et al., 2017), we compared the percentage of children in our sample who scored within normal limits on our measures of externalizing behaviors (the ECBI and BASC-3-Externalizing Problems Composite) and internalizing behaviors (the BASC-3-Internalizing Problems Composite) at pre-treatment, post-treatment, and follow-up. Because such comparisons comprised paired dichotomous measures, we then utilized McNemar’s test to examine whether these differences in percentages were statistically significant (Pembury Smith and Ruxton, 2020). Critical *p*-values were Bonferroni-adjusted, as described above, to account for Type I error inflation that may occur due to multiple comparisons (Bonferroni, 1936).

Third, we investigated which pre-treatment, demographic, and socioeconomic characteristics predicted treatment retention and outcomes in our 18-week intervention model. We conducted this investigation in a two step-process. First, we examined correlations between each of our 29 predictors and 6 treatment outcomes (ECBI, BASC Externalizing Problems Composite, BASC Internalizing Problems Composite, compliance rate, “Do” skills, “Avoid” skills) at both post-treatment and the 1 month follow-up. Correlations were also examined between each of our 29 predictors and 2 retention outcomes (completion of CDI and treatment). Second, for each treatment outcome or retention variable, predictors found to be significant in correlation analyses were included together as simultaneous predictors of the treatment outcome in regression-based path analyses (Kline, 2011). This two-step process was done to avoid over-interpretation of significant bivariate correlations between a predictor and outcome that did not control for the effects of other significant predictors.

#### 2.4.1. Missing data analyses

Families that completed post-treatment and follow-up assessments were compared to families who did not on all pre-treatment measures of child externalizing and internalizing behavior, child compliance, and caregiver skills. Pre-treatment variables did not differ between those who completed post-treatment assessments and those who did not. Additionally, pre-treatment variables did not differ between families who completed follow-up assessments and those who did not, with two exceptions. Caregivers who completed follow-up assessments demonstrated one more “Do” skill at pre-treatment [*M_Follow-UpCompleters_* = 5.16, *M_Follow-UpNon-completers_* = 4.30; *t*(457) = −2.09, *p* = 0.04], and three more “Avoid” skills at pre-treatment [*M_Follow-UpCompleters_* = 30.35, *M_Follow-UpNon-completers_* = 27.35; *t*(457) = −1.95, *p* = 0.05]. Given that significant differences were found on only 2 of 14 possible pre-treatment variables, and these differences were small, it does not appear that there were problematic systematic differences between those who completed and those who did not complete post-assessment and follow-up data. Consequently, such data missingness is not likely to substantively impact study results. Nevertheless, in line with best practice, full information maximum likelihood estimation procedures were used to ensure that all families, even those with partially missing data, were included in the regression-based path analyses predicting parenting skills and child behavioral outcomes described below (Kline, 2011).

## 3. Results

### 3.1. Time-limited PCIT model family outcomes

Table 2 reports the changes in child and caregiver outcomes from pre-treatment, to post-treatment, to the 1-month follow-up. Table 2 provides preliminary evidence that the 18-week model improves caregiver and child outcomes. Regarding child externalizing behavior, average ECBI Intensity raw scores dropped 57 points from pre-treatment to post-treatment (*d =* 1.79), and this drop was maintained at the 1-month follow-up (Table 2). Notably, even by mid-treatment, average ECBI scores (*M* = 111.36, *SD* = 33.21) had already dropped from the clinically elevated range (i.e., above 130) into well within normal limits, even reaching graduation criteria (i.e., at or below 114). Moreover, these differences from pre-treatment to mid-treatment ECBI scores were statistically significant [*t*(334) = 20.08, *p* < 0.01] and large in effect size (*d* = 1.09).

Similarly, child compliance rates increased from approximately 53% to approximately 75% at post-treatment (approximately 0.4 standard deviations; Table 2), and this improvement was maintained at the 1-month follow-up. Moreover, *T*-scores dropped approximately 10 points (approximately 1.2 standard deviations) on the BASC Externalizing Problems Composite and approximately 7 points (approximately 0.9 standard deviations) on the BASC Internalizing Problems Composite from pre-treatment to post-treatment. All of these effects were statistically significant and considered large in magnitude (*d* > 0.8; Cohen, 1988) except for the child compliance rate effect size, which was approximately medium in magnitude (*d* = 0.5; Cohen, 1988).

Caregiver outcomes demonstrated similarly large improvements from pre-treatment to post-treatment and pre-treatment to 1-month follow-up (Table 2). Caregiver “Do” skills, as demonstrated in a 5-min observation at each time point, increased by about 20 skills (1.9 standard deviations) from pre-treatment to post-treatment, and this was maintained at follow-up (Table 2). Caregiver “Avoid” skills decreased by about 22 skills (1.4 standard deviations) from pre-treatment to post-treatment and pre-treatment to follow-up, and both of these effects were considered statistically significant and large in magnitude (Cohen, 1988).

### 3.2. Time-limited PCIT model impact on clinically significant changes in child behavior

Table 3 reports the percentage of children in the sample who scored within normal limits on measures of externalizing (i.e., ECBI and BASC Externalizing Problems Composite) and internalizing (BASC Internalizing Problems Composite) behavior at pre-treatment, as well as post-treatment and at the 1-month follow-up. Across all three measures, Table 3 provides evidence that the 18-week model produced clinically significant changes in the vast majority of families who completed treatment. Specifically, the percentage of the sample within normal limits on the ECBI increased from approximately 30% at pre-treatment to approximately 90% at the 1-month follow-up time point (Table 3). Similarly, the percentage of the sample that met ECBI graduation criteria (<= 114 ECBI Intensity Score) increased from approximately 15% at pre-treatment to approximately 80% at the 1-month follow-up (Table 3). Both differences were statistically significant. Furthermore, of those children who had clinically elevated ECBI scores at pre-treatment, 63.4% were within normal limits by mid-treatment (and 46.38% had reached graduation criteria), 89.59% were within normal limits at post-treatment (and 78.28% had reached graduation criteria), and 86.09% were within normal limits at 1-month follow-up (and 72.19% had reached graduation criteria).

Similar results emerged when BASC Externalizing and BASC Internalizing Problems Composite scores were examined (Table 3). The percentage of the sample scoring within normal limits on the BASC Externalizing and BASC Internalizing Problems Composite scores rose from 42% and 65% at pre-treatment, respectively, to 82% and 84% at post-treatment, respectively. Both differences were also statistically significant. Thus, of those children who had at-risk or clinically elevated BASC Externalizing Problems Composite t-scores at pre-treatment, 69.30% fell within normal limits at post-treatment. Of those children who had at-risk or clinically elevated BASC Internalizing Problems t-scores at pre-treatment, 60.32% fell within normal limits at post-treatment.

### 3.3. Identifying predictors of treatment retention and outcomes

Third, we investigated what family and treatment factors (i.e., sociodemographic, treatment format, pre-treatment variables, treatment process variables) predicted retention and treatment outcomes and describe predictors of each outcome below.

#### 3.3.1. Predictors of child externalizing problems

Correlational analyses indicated that there were five significant predictors of post-treatment and follow-up ECBI scores (Table 4). However, when the five significant predictors of post-treatment ECBI scores were included in a single regression-based path model, only one effect emerged as significant (Table 5). Specifically, higher pre-treatment ECBI scores predicted higher post-treatment ECBI scores, even when all other predictors found significant in correlational analyses were controlled for. Similarly, of the five predictors of ECBI scores at 1-month follow-up that were significant in correlational analyses, only two remained significant in the regression-based path model. Higher pre-treatment ECBI Intensity scores predicted higher follow-up ECBI Intensity scores. Being from a Haitian or English-speaking Carribean caregiver family predicted lower follow-up ECBI Intensity scores.

**Table 4:** Correlations between predictors and key outcome variables.

	ECBI		Compliance Rate		Do Skills		Avoid Skills		BASC-Ext	BASC-Int	Completion	
Predictor	Post	FUp	Post	FUp	Post	FUp	Post	FUp	Post	Post	CDI	PCIT
Child gender	−0.04	0.02	0.01	0.07	0.07	0.02	−0.11	0.03	**0.17**	**0.19**	0.06	0.06
Child age (Months)	−0.06	0.00	−0.03	0.11	**−0.20**	**−0.24**	0.05	0.00	−0.12	−0.03	−0.03	−0.03
Caregiver education	0.00	−0.03	0.08	−0.05	0.10	0.13	0.00	−0.07	0.00	−0.13	**0.23**	**0.30**
Caregiver ethnicity: Hispanic	−0.03	0.04	**−0.13**	−0.08	−0.06	**−0.14**	0.02	0.04	**−0.15**	−0.05	0.03	0.04
Caregiver Race: Native American	0.07	0.05	**−0.12**	0.05	−0.09	0.05	0.02	0.00	**0.17**	0.09	−0.08	−0.05
Caregiver Race: Asian	−0.07	−0.02	0.10	0.07	0.00	−0.02	−0.05	−0.05	−0.08	−0.05	0.06	0.08
Caregiver Race: Black	0.08	−0.02	0.00	0.12	**−0.14**	**−0.13**	0.09	0.15	0.05	0.05	**−0.14**	**−0.20**
Caregiver Race: Pacific Islander	−0.02	0.01	**−0.12**	0.05	−0.09	0.05	−0.04	−0.04	−0.05	0.01	0.03	0.03
Caregiver Race: White	0.03	0.12	−0.08	**−0.16**	0.04	−0.03	−0.01	−0.07	−0.01	−0.13	**0.10**	**0.14**
Caregiver Race: Other	−0.03	−0.08	0.05	0.05	0.02	0.00	0.06	0.09	−0.05	**0.14**	0.05	0.03
Caregiver Race: Multiracial	−0.03	−0.02	0.07	0.00	0.05	0.10	−0.07	−0.12	0.04	−0.02	0.06	0.04
Caregiver Race: Haitian or English-Speaking Caribbean	−0.09	**−0.17**	0.01	0.07	0.06	0.07	0.00	0.11	−0.03	0.10	0.02	0.01
HUD Low-Income	**0.16**	0.04	0.00	−0.06	−0.01	−0.19	−0.11	0.04	0.16	**0.21**	**−0.15**	**−0.24**
Services in English	−0.03	−0.01	−0.01	0.07	−0.02	0.08	−0.10	−0.13	**0.16**	−0.09	0.04	0.08
Services in Spanish	0.03	0.06	−0.04	−0.04	0.04	0.06	0.03	0.06	−0.16	**0.17**	−0.08	**−0.11**
Services in English and Spanish	−0.03	−0.05	0.05	−0.05	−0.03	**−0.19**	**0.14**	0.11	−0.09	**−0.15**	0.04	0.04
Services in Creole and English	0.08	N/A	0.06	N/A	0.03	N/A	−0.04	N/A	**0.16**	0.13	0.05	−0.03
2 Caregivers Participating	−0.01	0.04	−0.03	0.06	**−0.13**	−0.05	0.02	−0.06	−0.02	−0.03	**0.15**	**0.16**
Randomized to Natural Helper Intervention	0.07	0.43	−0.26	**−0.53**	0.06	**0.51**	−0.11	−0.15	0.31	−0.06	−0.19	−0.13
Received CFI	−0.03	0.07	−0.07	−0.01	**−0.23**	−0.10	**0.17**	**0.20**	0.01	0.01	−0.07	−0.04
Participated During COVID-19	0.00	−0.01	0.08	**0.16**	**−0.25**	−0.12	**0.17**	**0.15**	**0.14**	0.02	−0.01	−0.01
Completed PCIT *via* telehealth	0.01	0.01	0.10	**0.20**	**−0.20**	−0.08	0.11	0.09	**0.14**	0.02	**0.10**	**0.11**
Pretreatment Do Skills	−0.01	−0.03	0.10	−0.05	**0.28**	**0.28**	−0.05	**−0.18**	0.02	0.04	0.06	**0.10**
Pretreatment Avoid Skills	0.00	−0.02	0.01	−0.04	**0.15**	0.13	**0.20**	**0.19**	0.01	−0.03	0.06	0.03
Pretreatment Child Compliance Rate (%)	0.02	−0.10	**0.15**	0.06	−0.10	**−0.18**	−0.01	0.10	0.02	0.02	0.01	−0.03
Pretreatment ECBI	**0.41**	**0.42**	−0.02	−0.06	−0.05	−0.05	0.04	0.11	**0.38**	**0.20**	−0.04	−0.05
Pretreatment PSI	**0.18**	**0.15**	0.02	−0.06	−0.01	−0.02	−0.03	−0.05	**0.30**	**0.22**	0.03	0.01
Pretreatment BASC-Ext	**0.33**	**0.25**	−0.01	−0.05	0.05	**0.20**	−0.10	−0.01	**0.68**	**0.31**	−0.03	−0.04
Pretreatment BASC-Int	**0.29**	**0.17**	−0.03	0.02	0.09	0.10	−0.06	−0.02	**0.26**	**0.70**	0.00	−0.03

HUD, Housing and Urban Development Criteria; CFI, Cultural Formulation Interview; IPCT, Internet-Based PCIT; ECBI, Eyberg Child Behavior Inventory Intensity Scale Raw Score; PSI, Parenting Stress Index Total Stress percentile score; BASC-Ext, Behavioral Assessment System for Children, Third Edition Externalizing Problems Composite *t*-score; BASC-Int, Behavioral Assessment System for Children, Third Edition Internalizing Problems Composite *t*-score; FUp, 1 Month Follow-Up; CDI, Child Directed Interaction; PCIT, Parent Child Interaction Therapy. Bolded values indicate correlations that were significant at *p* < 0.05. N/A, Among those that completed follow-up reports, none received services in Creole and English.

**Table 5:** Regression models predicting key outcome variables from predictors emerging as significant in previous correlation analyses.

Predictor	β or OR	SE	*p*
**Predictors of ECBI Post-Treatment (*R^2^* = 0.21)**			
HUD Low-Income	0.10	0.07	0.15
Pretreatment ECBI	**0.33**	**0.07**	**<0.01**
Pretreatment PSI	−0.02	0.07	0.83
Pretreatment BASC-Ext	0.11	0.08	0.19
Pretreatment BASC-Int	0.08	0.10	0.40
**Predictors of ECBI 1 Month Follow-Up (*R^2^* = 0.30)**			
Caregiver Race: Haitian or English-Speaking Caribbean	**−0.26**	**0.05**	**<0.01**
Pretreatment ECBI	**0.39**	**0.08**	**<0.01**
Pretreatment PSI	0.00	0.08	0.97
Pretreatment BASC-Ext	0.09	0.09	0.33
Pretreatment BASC-Int	0.05	0.09	0.59
**Predictors of Compliance Rate Post-Treatment (*R^2^* = 0.08)**			
Caregiver Ethnicity: Hispanic	**−0.14**	**0.05**	**<0.01**
Caregiver Race: Native American	**−0.15**	**0.05**	**<0.01**
Caregiver Race: Pacific Islander	**−0.08**	**0.03**	**0.05**
Pretreatment Child Compliance Rate (%)	**0.17**	**0.06**	**0.01**
**Predictors of Compliance Rate 1 Month Follow-Up (*R^2^* = 0.30)**			
Caregiver Race: White	**−0.27**	**0.07**	**<0.01**
Randomized to Natural Helper Intervention	−0.47	0.24	0.06
Participated During COVID-19	−0.20	0.12	0.10
Completed IPCIT	**0.40**	**0.13**	**<0.01**
Pretreatment Child Compliance Rate (%)	−0.05	0.09	0.60
**Predictors of Do Skills Post-Treatment (*R^2^* = 0.24)**			
Child Age (Months)	−0.07	0.06	0.29
Caregiver Race: Black	**−0.19**	**0.07**	**<0.01**
2 Caregivers Participating	**−0.15**	**0.05**	**<0.01**
Received CFI	**−0.17**	**0.06**	**<0.01**
Participated During COVID-19	**−0.43**	**0.19**	**0.02**
Completed IPCIT	0.27	0.19	0.17
Pretreatment Do Skills	**0.21**	**0.06**	**<0.01**
Pretreatment Avoid Skills	0.07	0.07	0.34
**Predictors of Do Skills 1 Month Follow-Up (*R^2^* = 0.24)**			
Child Age (Months)	−0.16	0.07	0.03
Caregiver Ethnicity: Hispanic	−0.12	0.13	0.35
Caregiver Race: Black	−0.23	0.11	0.04
Services in English & Spanish	**−0.14**	**0.05**	**<0.01**
Randomized to Natural Helper Intervention	−0.04	0.36	0.91
Pretreatment Do Skills	**0.20**	**0.08**	**0.01**
Pretreatment Child Compliance Rate (%)	**−0.18**	**0.06**	**<0.01**
Pretreatment BASC-Ext	0.19	0.10	0.07
**Predictors of Avoid Skills Post-Treatment (*R^2^* = 0.11)**			
Services in English and Spanish	0.14	0.07	0.06
Received CFI	0.13	0.08	0.10
Participated During COVID-19	0.10	0.06	0.13
Pretreatment Avoid Skills	**0.21**	**0.08**	**<0.01**
**Predictors of Avoid Skills 1 Month Follow-Up (*R^2^* = 0.14)**			
Received CFI	**0.18**	**0.09**	**0.04**
Participated During COVID-19	0.09	0.07	0.19
Pretreatment Do Skills	**−0.23**	**0.04**	**<0.01**
Pretreatment Avoid Skills	**0.23**	**0.09**	**0.01**
**Predictors of BASC-Externalizing Subscale Post-Treatment (*R^2^* = 0.51)**			
Child Gender	0.01	0.05	0.81
Caregiver Ethnicity: Hispanic	**−0.14**	**0.06**	**0.01**
Caregiver Race: Native American	**0.11**	**0.04**	**<0.01**
Services in English	−0.05	0.07	0.46
Services in Creole & English	0.05	0.05	0.26
Participated During COVID-19	−0.05	0.14	0.69
Completed IPCIT	0.09	0.14	0.53
Pretreatment ECBI	0.04	0.06	0.58
Pretreatment PSI	0.06	0.05	0.24
Pretreatment BASC-Ext	**0.66**	**0.06**	**<0.01**
Pretreatment BASC-Int	−0.10	0.08	0.18
**Predictors of BASC-Internalizing Subscale Post-Treatment (*R^2^* = 0.52)**			
Child Gender	0.03	0.05	0.62
Caregiver Race: Other	0.02	0.06	0.81
HUD Low-Income	0.05	0.06	0.38
Services in Spanish	0.06	0.07	0.36
Services in English & Spanish	−0.08	0.05	0.11
Pretreatment ECBI	−0.09	0.06	0.11
Pretreatment PSI	0.03	0.05	0.57
Pretreatment BASC-Ext	0.01	0.08	0.89
Pretreatment BASC-Int	**0.71**	**0.06**	**<0.01**
**Predictors of Completion of the Child Directed Interaction Phase (*R^2^* = 0.13)**			
Caregiver Education	**1.23**	**0.11**	**0.03**
Caregiver Race: Black	**0.51**	**0.21**	**0.02**
Caregiver Race: White	0.93	0.28	0.79
HUD Low-Income	0.57	0.23	0.06
2 Caregivers Participating	1.74	0.49	0.13
Completed IPCIT	1.26	0.29	0.38
**Predictors of Completion Parent Child Interaction Therapy Treatment (*R^2^* = 0.19)**			
Caregiver Education	**1.30**	**0.11**	**<0.01**
Caregiver Race: Black	**0.30**	**0.13**	**<0.01**
Caregiver Race: White	0.83	0.23	0.46
HUD Low-Income	**0.55**	**0.20**	**0.03**
Services in Spanish	0.70	0.19	0.11
2 Caregivers Participating	1.46	0.36	0.20
Completed IPCIT	1.24	0.26	0.37
Pretreatment Do Skills	1.04	0.03	0.10

HUD, Housing and Urban Development Criteria; CFI, Cultural Formulation Interview; IPCT, Internet-Based PCIT; ECBI, Eyberg Child Behavior Inventory Intensity Scale Raw Score; PSI, Parenting Stress Index Total Stress percentile score; BASC-Ext, Behavioral Assessment System for Children, Third Edition Externalizing Problems Composite *t*-score; BASC-Int, Behavioral Assessment System for Children, Third Edition Internalizing Problems Composite *t*-score; CDI, Child Directed Interaction; PCIT, Parent Child Interaction Therapy. Bolded values indicate parameters that were significant at *p* < 0.05.

Correlational analyses revealed 11 significant predictors of child BASC Externalizing Problems Composite Scale scores at post-treatment (Table 4). When the effects of those 11 post-treatment predictors were estimated simultaneously in regression-based path analyses, 3 predictors remained significant (Table 5). Caregivers who identified as Native American reported higher BASC Externalizing Problems Composite scores. Caregivers who identified as Hispanic reported lower BASC Externalizing Problems Composite scores. Additionally, higher pre-treatment BASC Externalizing Problems Composite scores predicted higher post-treatment Externalizing Problems Composite scores.

#### 3.3.2. Predictors of child internalizing problems

Correlational analyses revealed nine significant predictors of child BASC Internalizing Problems Composite scores at post-treatment (Table 4). When the effects of those nine post-treatment predictors were estimated simultaneously in regression-based path analyses, one predictor remained significant (Table 5). Higher pre-treatment BASC Internalizing Problems Composite scores predicted higher post-treatment BASC Internalizing Problems.

#### 3.3.3. Predictors of child compliance

Correlational analyses revealed four significant predictors of child compliance rate at post-treatment and five significant predictors of child compliance rate at follow-up (Table 4). When the effects of those four post-treatment predictors were estimated simultaneously in regression-based path analyses, all four predictors remained significant (Table 5). Specifically, children of caregivers who identified as Hispanic, Native American, or Pacific Islander demonstrated lower rates of post-treatment child compliance. In addition, higher pre-treatment child-compliance rates predicted higher post-treatment child-compliance rates (Table 5).

When the effects of the five follow-up predictors were estimated simultaneously, two predictors remained significant. Caregivers who identified as White reported lower rates of child compliance at follow-up. Children who completed treatment using telehealth (as opposed to in-person) demonstrated higher rates of follow-up compliance (Table 5).

#### 3.3.4. Predictors of caregiver “Do” skills

Correlational analyses revealed eight significant predictors of caregiver “Do” skills at post-treatment and follow-up (Table 4). When the effects of those eight post-treatment predictors were estimated simultaneously in regression-based path analyses, five predictors remained significant (Table 5). Specifically, caregivers who identified as Black, were part of a family where two caregivers participated, received the Cultural Formulation Interview, or participated in treatment during COVID-19 demonstrated fewer post-treatment “Do” skills. Caregivers who demonstrated more pre-treatment “Do” skills demonstrated more post-treatment “Do” skills (Table 5).

When the effects of the eight follow-up predictors were estimated simultaneously, three predictors remained significant. Caregivers who received services in English and Spanish and whose children demonstrated lower pre-treatment compliance rates demonstrated fewer “Do” skills at follow-up. Caregivers who demonstrated more pre-treatment “Do” skills demonstrated more follow-up “Do” skills (Table 5).

#### 3.3.5. Predictors of caregiver “Avoid” skills

Correlational analyses revealed five significant predictors of caregiver “Avoid” skills at post-treatment and follow-up (Table 4). When the effects of those four post-treatment predictors were estimated simultaneously in regression-based path analyses, one predictor remained significant. Caregivers who demonstrated more pre-treatment “Avoid” skills demonstrated more post-treatment “Avoid” skills (Table 5).

When the effects of the four follow-up predictors were estimated simultaneously, three remained significant. Caregivers who received the Cultural Formulation Interview and caregivers who demonstrated more pre-treatment “Avoid” skills demonstrated more “Avoid” skills at follow-up. In contrast, caregivers who demonstrated more pre-treatment “Do” skills demonstrated fewer “Avoid” skills at follow-up.

#### 3.3.6. Predictors of treatment completion

Correlational analyses revealed six significant predictors of CDI completion (Table 4). When the effects of those six predictors were estimated simultaneously in regression-based path analyses, two predictors remained significant (Table 5). Families with caregivers who identified as Black were less likely to complete the CDI phase, and families with caregivers who reported higher levels of education were more likely to complete the CDI phase (Table 5).

Correlational analyses revealed eight significant predictors of treatment completion (Table 4). When the effects of those eight predictors were estimated simultaneously in regression-based path analyses, three predictors remained significant (Table 5). Families with caregivers who identified as Black or who met the HUD low-income criteria were less likely to complete treatment, and families with caregivers who reported higher levels of education were more likely to complete treatment (Table 5).

## 4. Discussion

Although numerous PCIT studies have established its effectiveness in enhancing parenting skills and reducing children’s disruptive behaviors, barriers to treatment engagement remain a significant concern, especially for minoritized families. In an effort to determine the extent that time-limited PCIT can reduce these barriers, the current study evaluated the overall effectiveness of an 18-week model of PCIT and examined predictors of retention and treatment outcomes. Overall, findings suggest that the 18-week PCIT model is an effective intervention for the majority of treatment completers.

Consistent with previous time-limited PCIT studies (Bagner and Eyberg, 2007; Bagner et al., 2010), our results indicate that (a) both child (i.e., externalizing behavior and internalizing behavior, child compliance) and caregiver (i.e., parenting skills) outcomes improved from pre-treatment to post-treatment in the 18-week model, (b) these improvements were maintained when measured at the 1-month follow-up, and (c) these improvements were, for the most part, large in effect size. With regard to clinically significant changes, approximately a third of the sample (30%) had ECBI intensity scores within normal limits at pre-treatment, the percentage of scores that fell within normal limits tripled (89.6%) at post-treatment, and these reductions were maintained at the 1-month follow-up. Notably, by the end of the CDI phase, approximately two thirds of the sample had ECBI intensity scores within normal limits. Moreover, meaningful reductions in externalizing behaviors were also reflected in BASC Externalizing Problems scores. The percentage of externalizing behaviors within the normal limits range doubled from pre-treatment (40%) to post-treatment (80%). Further, a large effect size was found for clinical reductions in internalizing symptoms (e.g., anxiety, depression) following treatment, which is remarkable as PCIT was not designed to address internalizing symptoms. These findings are consistent with prior evaluations of PCIT (e.g., Choate et al., 2005; Chase and Eyberg, 2008; Carpenter et al., 2014), highlighting how the effects of PCIT (i.e., improved communication, positive attention, caregiver consistency) may incidentally break negative caregiver-child reinforcement cycles (e.g., accommodation, avoidance) that maintain internalizing behaviors (Settipani et al., 2013).

When examining predictors of treatment retention and child and caregiver outcomes, it appears that outcomes in the 18-week PCIT model were, by and large, equitable across ethnicity, race, socioeconomic status, language of service delivery, and other environmental circumstances. Of the 348 total associations between demographic predictors and post-treatment and follow-up outcomes explored, only 30 (9%) were significant in both zero-order correlation and regression frameworks. However, in the relatively rare instances where inequity in outcomes was identified, such inequities appeared to emerge most often due to (in descending order of breadth of outcomes affected), identification as a Black caregiver, identification as a Native American caregiver, use of the CFI, and level of caregiver education.

Being a Black caregiver (10% of sample) was predictive of fewer observed “Do” skills across assessment time points, as well as of reduced treatment completion rates. This finding may be partially explained by the fact that using “Do” skills, which reflect an authoritative parenting style, may feel inconsistent with the need for respect from children in cultures where more authoritarian parenting styles have been historically practiced. This potential mismatch between perceived expected parenting style (authoritative vs. authoritarian) may partially contribute to fewer observed *Do Skills* and reduced treatment retention. Additionally, a recent examination showed that the language standards used for the DPICS-IV, which was validated with predominantly White families, do not accurately capture the delivery of positive attention when speaking African American Vernacular English (AAVE), which may thereby falsely conflate the number of “Do” skills with “amount of positive attention (Chavez et al., 2021).”

Being a Native American caregiver (0.63% of the entire sample, which is reflective of the community where services were provided) was predictive of lower rates of child compliance and higher child externalizing behaviors at post-treatment. One possible explanation may be that practices taught and reinforced in PCIT may have brought heightened caregiver awareness to child behavior deemed negative by United States majority culture that may not necessarily be perceived as negative or in need of modification in Native American culture (Menakem, 2017). Additionally, while some families received the CFI, it is not known the extent that therapists integrated the values of Native American families into service provision and/or tailored services to be consistent with family’s values and/or perspectives about treatment, as outlined in the Honoring Children, Making Relatives cultural translation of PCIT for Native American families (Big Foot and Funderburk, 2011). This model of PCIT encourages modifications such as shifting to slower language cadences in parent–child interactions and coaching that feels more natural to families, increasing DPICS coding length from 5 to 7 min, and increasing inclusion of words that count as labeled praise that emphasize family (e.g., little grandpa; Big Foot and Funderburk, 2011). However, given the small Native American sample in this study, these results should be interpreted with caution.

Surprisingly, use of the Cultural Formulation Interview (CFI) was associated with lower post-treatment “Do” skills and higher follow-up “Avoid” skills. On the surface, this may appear to indicate that the CFI worked against its intended goal (to further engage families in treatment). However, the CFI was not predictive of post-treatment or follow-up child behavior outcomes, nor did it predict treatment completion.

Consistent with other PMT studies (Bagner and Graziano, 2013; Chacko et al., 2016) caregivers with lower levels of education were less likely to complete treatment. Notably, over two thirds of families reported child behaviors within normal limits by mid-treatment. Nevertheless, 18 weeks of treatment may still be experienced as a strenuous commitment for families with lower levels of education, who may be balancing several other demands and stressors (e.g., on-demand work shifts) and thus may benefit from shorter treatment durations. For example, prior research has demonstrated that families who receive at least four CDI sessions or complete briefer models of PCIT (e.g., 4–7 sessions) still demonstrate significant behavioral improvements (Berkovits et al., 2010; Lieneman et al., 2019; Timmer et al., 2021; Hawk et al., 2022). These findings suggest family-centered planning regarding preferred duration of services may serve as a potential solution for increased treatment retention, as opposed to making one PCIT model fit all families.

### 4.1. Strengths, limitations, and future directions

It is important to note several strengths of the current study. This study includes the most diverse (approximately 82% of caregivers identified as being a member of a racial and/or ethnic minority group) and largest single-study sample of time-limited PCIT families to date. Time-limited PCIT was shown to significantly reduce children’s externalizing and internalizing behaviors and increase effective parenting skills in the vast majority of treatment completers. To further understanding of PCIT retention for predominantly minoritized families, we simultaneously examined the impact of sociodemographic and treatment format, process, and pretreatment variables on treatment outcomes and retention. A limited set of factors (comprising only 9% of the variables) were discovered to exert a discernible influence on treatment outcomes and retention rates. This finding indicates that time-limited PCIT exhibits a quantifiable impact in advancing the progress towards achieving treatment equity.

Several study limitations also warrant discussion. First, while this study was conducted with a sample that generally mirrored the demographic composition of the community where services were delivered, this sample may be more representative of families with higher levels of education than the general educational levels of the community (over 57% of families included had achieved at least a Bachelor’s degree or higher compared to approximately 30% in the community). However, despite serving caregivers with higher education, approximately 50% of families who reported income were categorized as having low income by HUD. Though the study included all available data by utilizing intention-to-treat analyses, rates of participation in the 1-month follow-up were low and almost certainly contributed to conservative estimates of parameters and *p*-values. Finally, 92.6% of caregivers in the current study were mothers, so we were unable to make statistically well-powered comparisons between changes in mother and father parenting skills and child developmental outcomes. Future studies can focus on capturing father involvement in this 18-week treatment modality, and study it’s unique effects on child behavioral outcomes. Examining fathers is especially important given the ways in which racism, the COVID-19 pandemic, and their pernicious effects can affect minoritized fathers’ parenting (Palkovitz and Fagan, 2021), and the fact that Black and Hispanic fathers’ early engagement in play and learning activities before their children are age 5 enhances child socioemotional and behavioral skills in middle childhood (Fagan and Cabrera, 2022).

Taken together, the current study has important clinical and research implications. First, overall results advocate for the time-limited model of PCIT being a viable standard treatment approach for predominantly minoritized families which shifts away from the more universally accepted approach of criteria-based model of PCIT that requires caregivers to demonstrate parenting skill proficiency and rate children’s disruptive behaviors within normal limits before treatment graduation. Indeed, our results demonstrating similar child and caregiver outcomes across a multitude of family and treatment factors (e.g., race, ethnicity, levels of education, and levels of income) further highlight how time-limited PCIT can reduce barriers to care. Importantly, a time-limited approach can provide a possible mechanism to partially address the shortage of trained mental health providers (Prinz and Sanders, 2007; Weisenmuller and Hilton, 2021), as it can create a more efficient case turnaround, consequently assisting in reducing health disparity by serving a larger number of families seeking PMT services. Future PCIT research with minoritized families should include compensated opportunities for collaboration and co-design of potential solutions for determining models of PCIT (e.g., family-centered decision making regarding duration of treatment) that can be the most effective in enhancing retention and outcomes for predominantly minoritized families. In addition, longer-term follow-up is needed to better understand how treatment gains are maintained over time (e.g., 6 months, 1 year).

## Data availability statement

The raw data supporting the conclusions of this article will be made available by the authors, without undue reservation.

## Ethics statement

The studies involving humans were approved by University of Miami Miller School of Medicine. The studies were conducted in accordance with the local legislation and institutional requirements. Written informed consent for participation in this study was provided by the participants’ legal guardians/next of kin.

## Author contributions

JJ, WR, AP, AW, ED, and DG designed the study. JJ, WR, and DG extracted and analyzed the data. JJ, WR, AP, JA, AW, RC, CD, JB, CS, and FC drafted the manuscript. NH and ED made essential comments and substantial revisions to the manuscript. All authors contributed to the article and approved the submitted version.
